# Light People: Professor Martin Booth spoke about adaptive optics and its applications

**DOI:** 10.1038/s41377-022-01001-5

**Published:** 2022-10-20

**Authors:** Jiahe Cui, Chao He, Wei Chang

**Affiliations:** 1 Department of Engineering Science, University of Oxford, 17 Parks Road, Oxford, OX1 3PJ UK; 2 Light Publishing Group, Changchun Institute of Optics, Fine Mechanics and Physics, Chinese Academy of Sciences, No. 3888 Dong Nan Hu Road, 130033 Changchun, China

**Keywords:** Adaptive optics, Imaging and sensing

## Abstract

Adaptive optics (AO), a technique originally introduced by astronomers to correct for optical distortions when looking at distant stars, now benefits the entire optics and photonics society. Prof. Martin Booth, who leads his research lab at the University of Oxford where he started his career, was one of the first people to take advantage of AO for microscopy. Since then, he has been continuously promoting the wide application of AO in all aspects of biological research and material science. As one of the few people who has witnessed the growth of AO in various communities, Prof. Martin Booth talks about their major differences, as well as their current challenges and future development. He discusses how AO can benefit emerging areas and the key challenges that may be faced. Prof. Martin Booth has also been making continuous efforts in removing the barriers of AO so that it can be promoted towards the wider community. Finally, he shares his experience from actively taking up different roles in education and in various societies and provides valuable advice to all early career researchers on the wisdom of being successful in their future careers. It is an honour for us to invite Prof. Martin Booth on this issue, and to learn from his inspirations, enthusiasm, and dedication.

## Abstract (web summary)

Prof. Martin Booth, as one of the first people to explore the practical use of adaptive optics in microscopy, shares with us his valuable experience and insight

## Graphical abstract

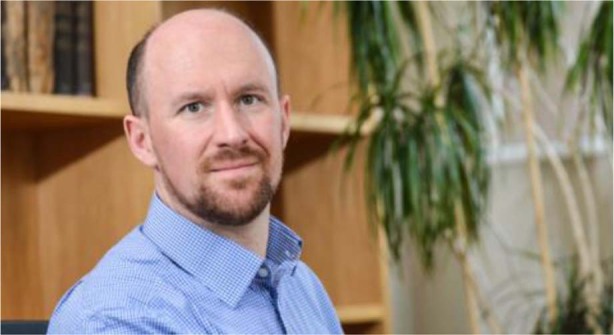

**Biography:** Prof. Martin Booth read for a degree in Engineering Science at Hertford College, Oxford, from 1993-7. His doctoral work in adaptive optics for confocal microscopy took place in the Department of Engineering Science at the University of Oxford from 1997-2001, during which time he was also a member of Jesus College. In 2001, Martin was elected to a Junior Research Fellowship at Christ Church and in 2003 was appointed a Royal Academy of Engineering/EPSRC Research Fellow. In 2007, he was awarded a five-year EPSRC Advanced Research Fellowship and was concurrently elected to a Hugh Price Fellowship at Jesus College. He became Professor of Engineering Science and Senior Research Fellow at Jesus College in 2014. He is now Deputy Head of Department, and also a fellow of Optica (formerly known as OSA), the Society of Photo-optical Instrumentation Engineers (SPIE), and the Institute of Physics (IOP). His current research interests centre on the development of new dynamic optical methods for applications ranging from biomedical imaging to laser-based manufacturing.

**Figure:**
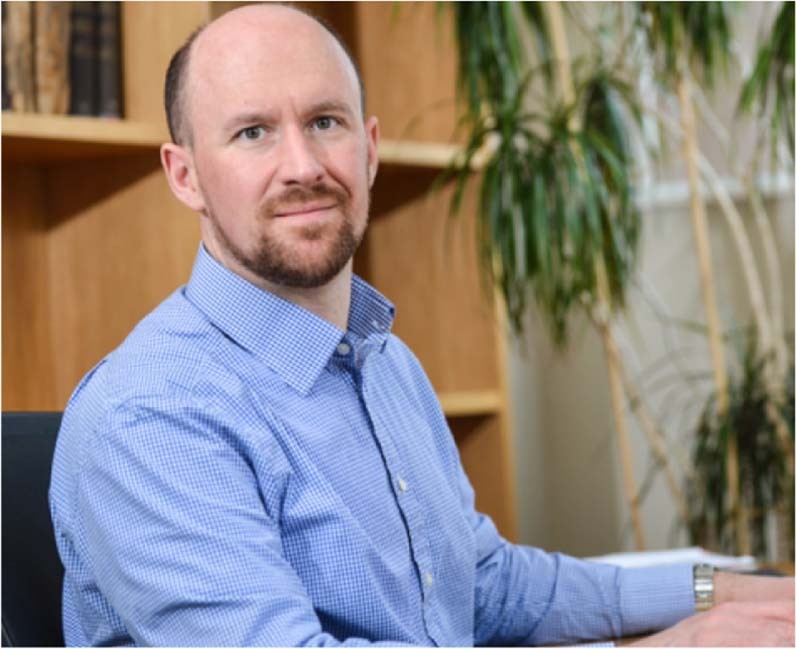

**Q1: AO is a powerful technique and is widely adopted in various research fields, with applications ranging in scale from astronomical telescopes to super-resolution microscopes. Can you talk about the differences in focus between AO in different fields and how they inherit and develop?**

AO was most famously developed for astronomical telescopes before being translated to other applications like ophthalmology and microscopy. The main differences in approach are due to the different scales of these applications. In astronomy, it is normal to have a large budget to produce one AO system for a telescope, whereas in the other applications, we are aiming towards systems that can be replicated many times for much lower cost. This means that while we can draw inspiration between the fields, the solutions we require are often very different.

**Figure:**
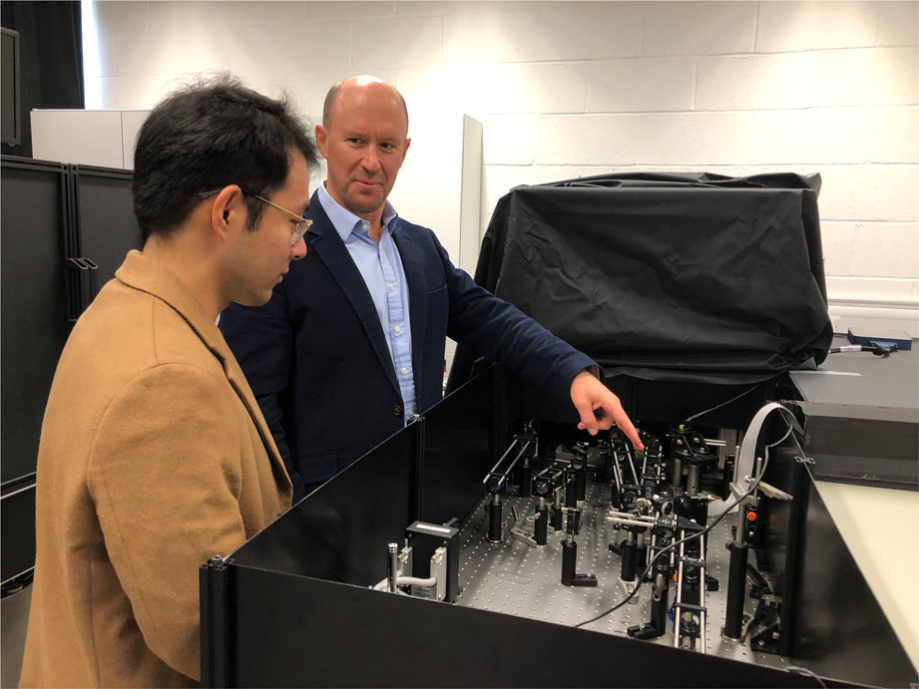
Prof. Martin Booth with Mr. Yifei Ma in his laboratory

**Q2: Your team has been continuously developing cutting-edge AO techniques in both sensor-based and sensorless regimes. Can you tell us about their major differences and key considerations during implementation? What do you think are the critical challenges of their future development?**

For our work in microscopy, we have been aiming towards solutions that are widely applicable across the many modalities of microscopes that might benefit from AO. Sensorless AO—that is, AO that does not require a wavefront sensor—is promising here, as the system has simpler hardware and can be used in imaging applications where it is difficult to use a wavefront sensor. The main challenge for these methods in the future is to generalise their operation so that they can be easily translated across different types of microscopes.

**Q3: As a pioneer of AO in microscopy, what challenges do you think AO is facing at its current stage? And what will be the major trends for its future development?**

The benefit of adaptive optics in microscopy has been widely shown in the laboratory. The main challenge now is to translate the technology into wider use. Ultimately, AO should be embedded inside the system and used seamlessly without user intervention. This will involve reductions in cost and size of the required equipment. It will also require the development of software and control algorithms that can be used reliably in an automated way.

**Q4: You have long been passionate about using AO for laser machining and have recently published papers in** ***Light: Science & Applications*** **(*LSA*) [On-chip beam rotators, polarizers, and adiabatic mode converters through low-loss waveguides with variable cross-sections]. Can you talk about the importance of AO in this field and its future prospects?**

Over the past years, we have made many advances in using adaptive optics for precision three-dimensional micro-fabrication. It is necessary for many applications to focus inside transparent materials. In these applications, aberration correction is required to overcome the effects of refraction at the surface, which causes the spreading of the focus and loss of precision. Our recent results in structured waveguides show great promise for a range of applications, for example, in the implementation of waveguide circuits for quantum optics applications.

**Figure:**
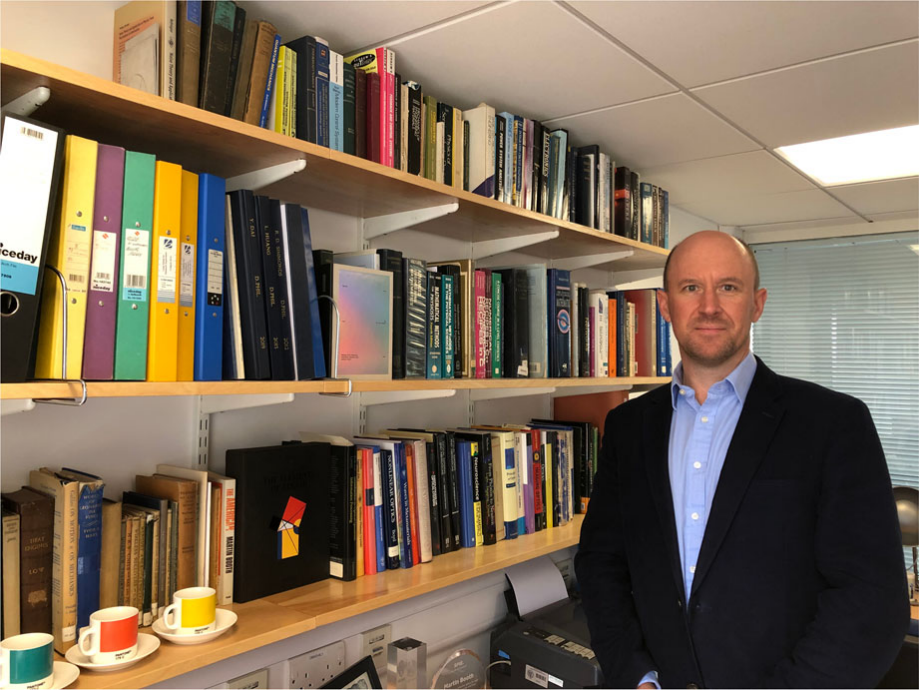
Prof. Martin Booth with his treasured book selection

**Q5: Your team has been implementing cutting-edge AO techniques in new fields, such as label-free microscope/micro-endoscope imaging for clinical diagnosis, amongst others. What potential benefits can AO bring to these fields? Could you please share with us some valuable experience?**

The main benefit of AO is to resolve smaller details and provide higher signal-to-noise ratio (SNR) during data acquisition. In many clinical applications, much benefit can be brought by visualising subcellular details and to identify potential biomarkers for non-invasive diagnosis. Enhancement in SNR also allows for faster acquisition of images, and thus makes it possible to provide real-time streaming in many clinical scenarios where it would be difficult to do so without.

**Q6: AO mainly focuses on the manipulation of phase. However, in many optical systems, such as certain super-resolution microscopes, polarisation aberrations play an even more crucial role, as incorrect polarisation states disrupt the interference at the focus and affect image resolution. Can you provide some insight on the development of adaptive correction for polarisation errors? What type of challenges might it face?**

Combined polarisation and phase correction—or vectorial AO—is an exciting new direction for adaptive optics. There are more degrees of freedom than for phase compensation alone, so there is more of a challenge. Moreover, the degrees of freedom are coupled, for example—it is not possible to correct polarisation states on their own without considering the phase compensation. This will require new ways to sense the vectorial aberrations, the adoption of methods for full generation of vectorial states, and the development of new algorithms for aberration control.

**Q7: You have recently published an advanced review paper [Polarisation optics for biomedical and clinical applications: a review]. In your opinion, what major problems can AO address in order to promote polarisation techniques for biomedical applications? Can you talk about its potential impact?**

There are many promising prospects in extending the use of polarisation for biomedical applications, particularly as it can provide label-free contrast. Combining this technology with AO will aid the use of such methods in three-dimensional specimens. There is also the prospect of using adaptive elements to simplify the design of polarimetric microscopes for more practical applications.

**Figure:**
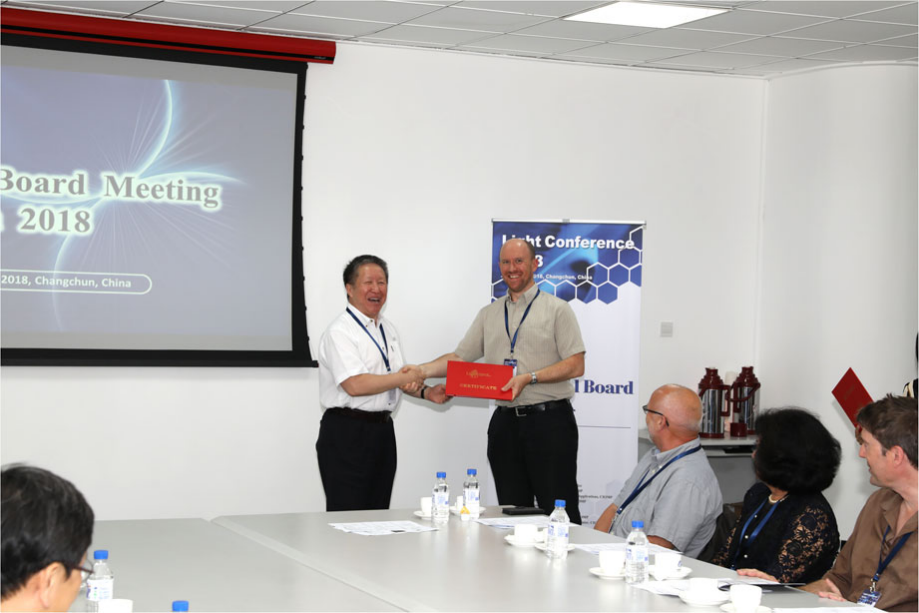
Prof. Jianlin Cao appointing Prof. Martin Booth as an Editor of *LSA*

**Q8: We have seen the AO tutorial website set up by you and your team. A lot of effort has been put into promoting the wide use of AO techniques, especially for those who are not experts in AO. What are the special focuses and how are contents laid out to accommodate non-specialists? Can you talk about its future prospect?**

The main barriers of using AO are not technical, but rather practical. There has been an overhead in effort required to incorporate AO technology into other methods. We therefore decided that we could help the adoption of this technology by providing tutorials and guidelines about AO to remove or reduce some of these barriers. We are also working towards being able to provide services and support to those who want to use AO. This approach is likely to be quicker and more cost effective to users than implementing AO in house.

**Q9: You are a fellow of Optica (formerly known as The Optical Society (OSA)), the Society of Photo-optical Instrumentation Engineers (SPIE), the Institute of Physics (IOP), as well as other important societies and organisations. Could you please share with us some experience of working in such associations?**

These professional societies play an important role in communication with others across the optics and photonics field. I have been a member of these societies since I was a doctoral student and they have provided many opportunities to interact with colleagues, whether through conferences, networking events or committees. I would encourage students to engage with these organisations to help in their own career development.

**Q10: As an editor of** ***LSA*, you have witnessed its birth, growth, and development. Recently, its new sister journals** ***Light: Advanced Manufacturing*** **and** ***eLight*** **have continuously published great works. What perspective and suggestions do you have for their future development?**

I think it is really important to have prominent journals that select articles based on their quality and their importance to the field, rather than other criteria. I believe that *LSA* has always been guided by this principle and this approach has led to its increasing respect in the field. It should continue to provide this service to the field of optics and photonics into the future.

**Figure:**
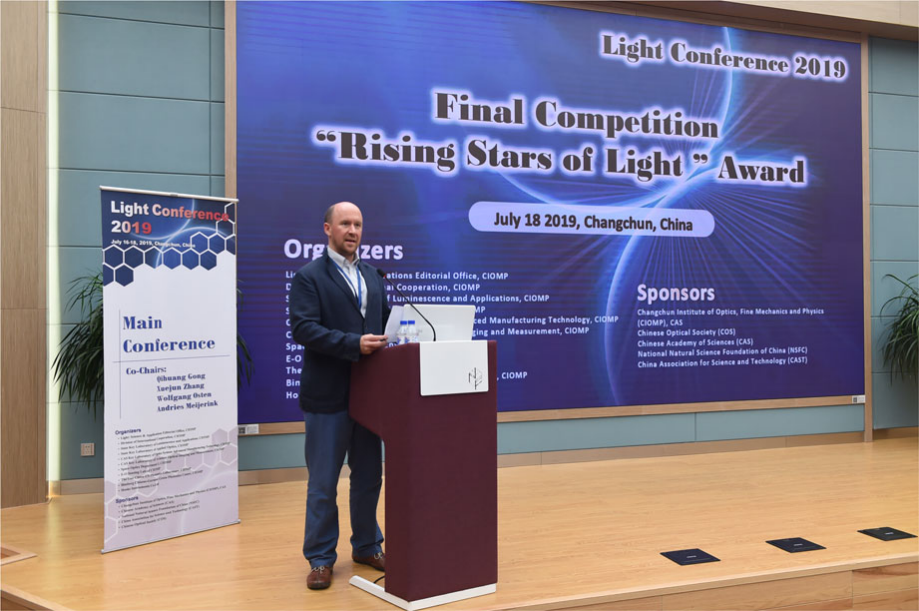
Prof. Martin Booth giving a speech for the ‘Rising Stars of Light’ Award at Light Conference 2019

**Q11: As far as we know, apart from microscopy you also have an excellent track record in areas of physics, biomedicine, etc. Would you consider yourself as an engineer, a physicist, or a mathematician?**

I have always been an engineer by training, so our work is driven by creating solutions to real technical problems. However, in order to do that we work across the range from optical theory through to commercialisation. Our research group therefore has expertise spanning engineering, physics, and mathematics, in addition to many other qualities and skills.

**Q12: You have always been a responsible scientist and a dedicated educator. What is the difference between delivering scientific content to different audiences? What general approaches do you take to optimally engage in different occasions?**

It is vital to remember that the most important person in these circumstances is the listener, viewer, or reader. You should always try to find where they are starting from in terms of understanding your subject and make sure you build up your story from that point. It is also important to be clear what messages you need to get across to the audience. If you cannot define a small number of core messages for a presentation, then it is difficult to make sure that the audience will learn what they need.

**Q13: You have a very large team of students and post-docs. When interviewing them, what qualities do you look for?**

I think that the most important skills are to be able to learn quickly and solve problems proactively. Many technical matters can be learned through reading and practical experience, so doing this efficiently is important. However, even more important is being able to apply that knowledge in novel ways. This is probably the most important skill for a research scientist.

**Q14: You have always encouraged young researchers to stand at the forefront of scientific research, to pursue what they love, and to have a great career. What advice would you have for them now, as well as for all early career researchers?**

It is tempting as an early career researcher to over-plan one’s career. It is of course important to think about future steps and how you might achieve them. However, it is possible to worry too much about detailed steps, rather than concentrating on the most important thing, which is to carry out high-quality research. Often the unexpected outcomes lead on to the most interesting and rewarding projects. Also, the most important publications often arise out of unplanned or redirected research. It would be a shame for the world to miss out on impactful research from early career scientists if they are constrained by conventional approaches.

**Figure:**
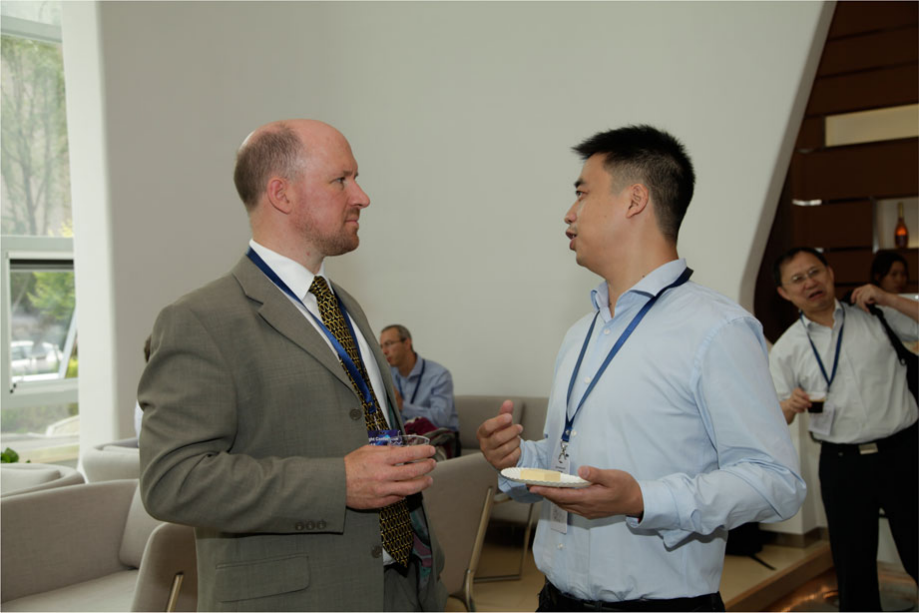
Prof. Martin Booth speaking to Prof. Min Qiu over conference coffee break

**Q15: It is challenging to stand at the forefront of scientific research. Could you please share your opinions with early career researchers on how to balance life and work?**

This is a challenge in any walk of life. We have to remember that scientific research is a creative process. It may require hard work, but it also requires good imagination. Often the best ideas come when not concentrating on the details. It is therefore essential to ensure that you can get away from the office or the lab. A change of scenery every so often can help a lot.

**Q16: As a successful researcher, has anyone had a major influence on you in your career? In what way?**

It is very difficult to name one person without naming many. I have certainly benefitted from working with a lot of excellent people, ranging from undergraduate students through to senior colleagues. In retrospect—and I only realised this long after leaving school—I was influenced at an early stage by my English literature teacher in high school. We had many fascinating lessons discussing a wide range of topics, but they rarely seemed to be about English literature. I now realise what he was really teaching us was critical thinking and how to challenge preconceptions. These skills are vital in scientific research, but they are not subject specific.

